# Be Wary of Hiccups: An Unusual Case of COVID-19

**DOI:** 10.7759/cureus.12974

**Published:** 2021-01-28

**Authors:** Sayed K Ali, Diana Muturi, Karishma Sharma

**Affiliations:** 1 Internal Medicine, Aga Khan University, Nairobi, KEN; 2 Medicine, Aga Khan University, Nairobi, KEN

**Keywords:** covid-19, hiccups, covid-19 pneumonia

## Abstract

Hiccups, involuntary contraction of the diaphragm and intercostal muscle followed by an abrupt closure of the glottis, are a bothersome symptom that can be caused by a variety of illnesses or medications. Hiccups that persist for more than 48 hours should raise the suspicion of an underlying cause. Pneumonias, especially caused by the novel coronavirus, have rarely been reported to trigger hiccups. To the best of our knowledge, we present the first case in sub-Saharan Africa of a patient presenting to our institution with persistent hiccups and no other objective signs suggestive of underlying pneumonia. His high-resolution CT was suggestive of coronavirus disease 2019 (COVID-19) and a polymerase chain reaction (PCR) test confirmed the diagnosis. Our case highlights the need for a thorough history and physical examination in patients presenting with hiccups and the need to include COVID-19 in the differential diagnosis in such patients.

## Introduction

Persistent hiccups can be a bothersome symptom that prompts patients to seek medical care. Most hiccups are self-limiting, but if they continue past 48 hours, an underlying cause should be sought. There have been a handful of case reports of underlying pneumonia triggering the development of hiccups. We present the first case reported in sub-Saharan Africa of a patient presenting with persistent hiccups for seven days and found to have underlying coronavirus disease 2019 (COVID-19) pneumonia. Our case highlights one of the less common manifestations of COVID-19 and the need for a thorough history and physical exam in all patients presenting with hiccups especially during the ongoing pandemic.

## Case presentation

A 65-year-old male with poorly controlled diabetes and hypertension presented to our institution with persistent hiccups for seven days. His symptoms had started abruptly while at home and when home remedies did not work, he chose to seek medical care at our facility. He denied any past surgical procedures but had gained significant weight after retiring a few years ago. He was a local farmer growing corn and beans and denied a history of smoking or alcohol use. He remained compliant with his metformin one gram twice a day and losartan 50 mg once a day but admitted to a poor diet. Apart from the persistent hiccups, he denied shortness of breath, cough, fever, belching, or a history of reflux disease.

On admission, his vital signs were within normal range with an oxygen saturation of 94% on room air. He was an obese male and looked tired with intermittent hiccups during the physical exam. His cardiovascular and abdominal exams were within normal limits, but his lung exam revealed crackles at the base bilaterally. The rest of his physical examination was normal.

**Figure 1:**
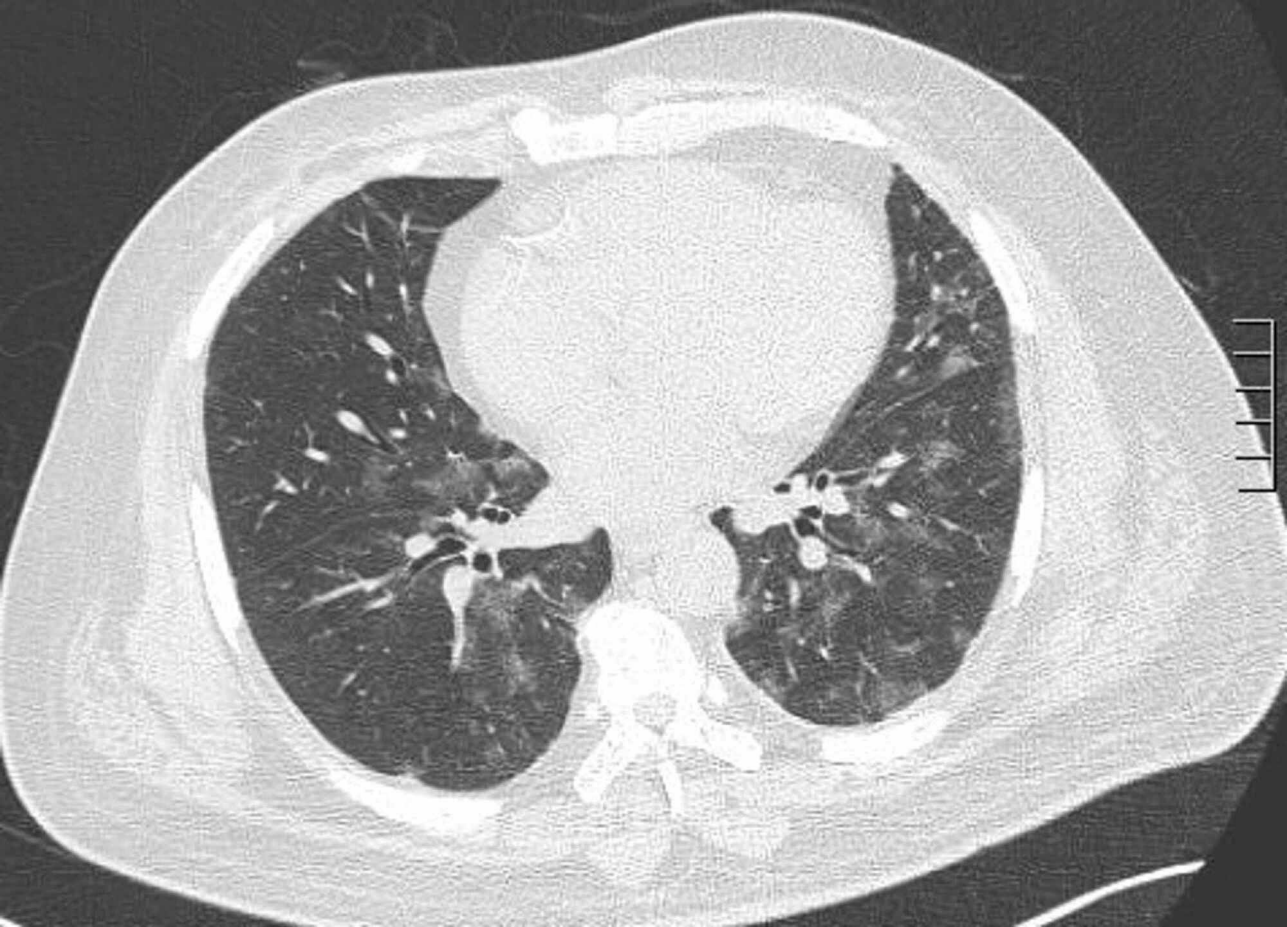
High-resolution CT showing peripheral glass-ground opacities

**Figure 2:**
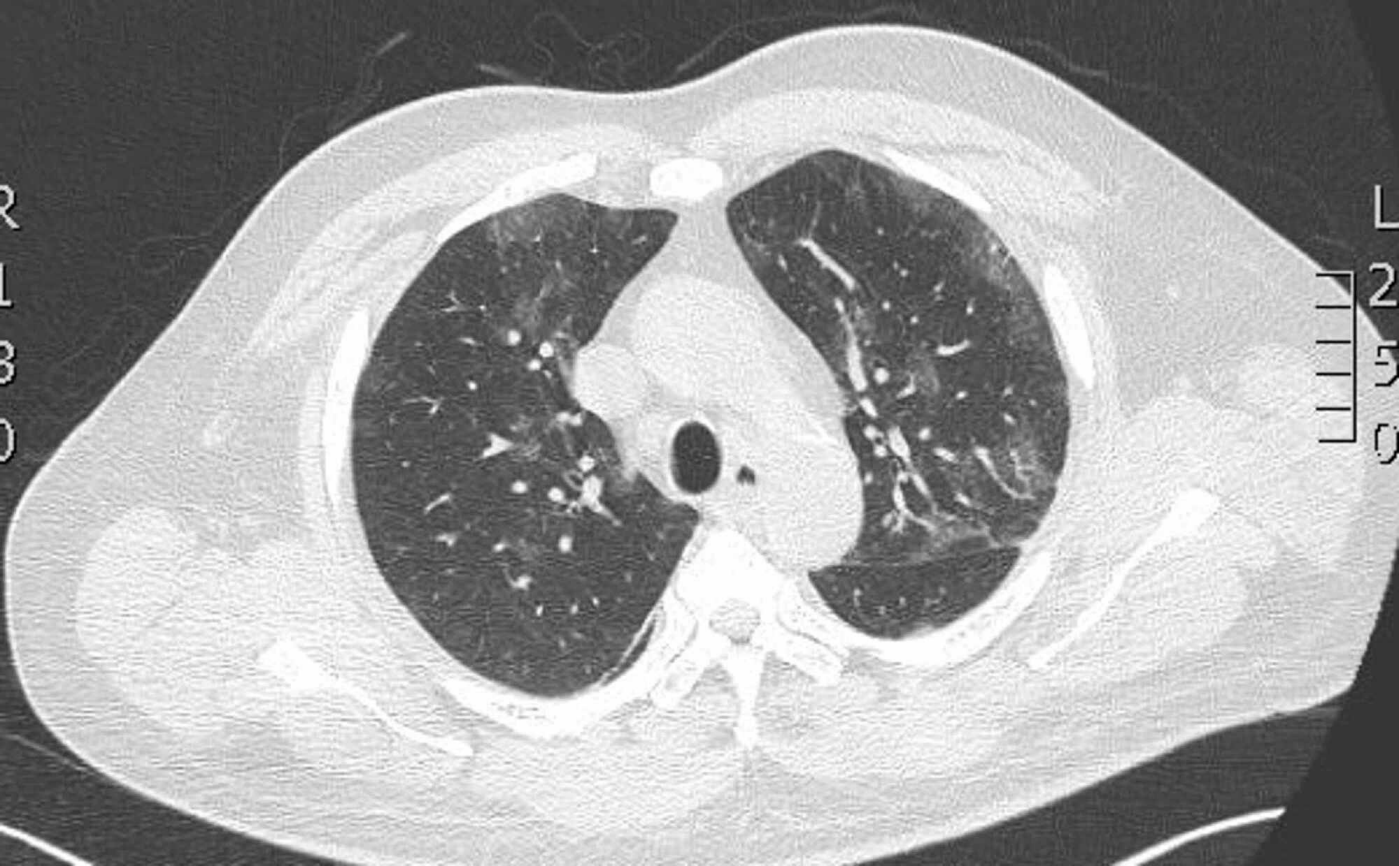
High-resolution CT showing peripheral glass-ground opacities

His complete metabolic panel was normal but his complete blood count showed a normal white count with a lymphopenia of 15.9%. His C-reactive protein (CRP) was elevated at 81 mg/L and d-dimer at 83 ug/ml. Due to his lung findings, a high-resolution CT was pursued that showed peripheral glass ground opacities typical for COVID-19 pneumonia (Figures 1-2). He was immediately transferred to the isolation ward and his COVID-19 polymerase chain reaction (PCR) returned positive within 24 hours of admission.

## Discussion

Singultus, the medical term for hiccups, originates from the Latin word singult that translates into a sob or gasp [1]. Hiccups are sounds produced by sudden involuntary contraction of the diaphragm and intercostal muscle followed by abrupt closure of the glottis [1,2]. Hiccups are a common occurrence in most mammals including cats, rats, horses, and humans, and are a reflex mechanism that might remove air from the stomach of suckling young [3]. More common in men, hiccups are often benign and self-limiting [4]. Persistent hiccups last for approximately 48 hours, while intractable hiccups usually last longer than one to two months. The exact mechanism of hiccups remains complex and poorly understood, but a reflex arc involving the peripheral phrenic, vagal, and sympathetic pathways, in addition to multiple neurotransmitters seem to play a role in hiccups [1-3].

The etiology of hiccups can range from central causes such as trauma and vascular diseases to various anti-Parkinson and antipsychotic drugs, reflux disease, myocardial infarction, various cancers, and electrolytes imbalances [2]. 

The first case of pneumonia presenting as “hiccough” was reported in Agra by Dr. Laha in 1951 where he describes a 68-year-old male with hiccups and left lower lung pneumonia [5]. In other isolated case reports, pneumonia, usually right-sided and involving the lower lung lobe, have presented as intractable hiccups. In such patients, the involvement of the lower lobes causing irritation of the phrenic nerve and its pericardial branches and the diaphragm are thought to be potential causes of hiccups [6,7].

To the best of our knowledge, there has been only one case of a patient who presented with persistent hiccups, fever, and mild tachycardia and was found to have COVID-19 [8]. Our case, the first of its kind in sub-Saharan Africa, remains unique as our patient presented with hiccups only and did not have any objective signs suggestive of underlying pneumonia.

Our patient was started on baclofen, a gamma-aminobutyric acid (GABA) receptor agonist which reduces dopamine release in turn interrupting the hiccups arc reflex. Baclofen is also known to relax skeletal muscles and transiently lower esophageal sphincter relaxations [9,10]. Our patient did not require any oxygen and hence was discharged home with self-isolation instructions. On follow up two weeks later, his hiccups had resolved and he felt much better. 

## Conclusions

A thorough history and good physical examination are crucial in patients presenting with persistent hiccups to help detect the underlying cause. In these times, COVID-19 should be included in the differential diagnosis of a patient presenting with persistent hiccups.
